# EPA guidance on cultural competence training: a critical update and meta-review

**DOI:** 10.1192/j.eurpsy.2026.12215

**Published:** 2026-05-13

**Authors:** Meryam Schouler-Ocak, Julia Zielke, Sofie Bäärnhielm, Ilaria Tarricone, Jerzy Samochowiec, Apostolos Veizis, Ahmet Tamer Aker, Pascal Markus Lemmer, Adrian Hagenguth, Dinesh Bhugra

**Affiliations:** 1 Department of Psychiatry and Neurosciences, Charité Campus Mitte, Charité – Universitätsmedizin Berlin AND Department of Psychiatry and Psychotherapy, Charité at St. Hedwig Hospital, Charité – Universitätsmedizin Berlin, Charite Universitatsmedizin Berlin, Germany; 2 Institute for Medical Sociology and Rehabilitation Science, Charité - Universitätsmedizin Berlin, Germany; 3 Karolinska University Hospital: Karolinska Universitetssjukhuset, Sweden; 4 Alma Mater Studiorum Universita di Bologna: Universita degli Studi di Bologna, Italy; 5 Pomeranian Medical University: Pomorski Uniwersytet Medyczny w Szczecinie, Poland; 6 Schering Plough Greece: MSD Greece, Greece; 7 Istanbul Bilgi Universitesi, Türkiye; 8 Charité - Universitätsmedizin Berlin, Germany; 9 King’s College London Department of Social Science Health and Medicine: King’s C, UK

**Keywords:** cultural competence, cultural humility, mental health, migration, training

## Abstract

**Background:**

Cultural competence training has become increasingly common in psychiatric practice across Europe and worldwide, supported by evidence demonstrating improvements in quality of care, clinical effectiveness, and patient satisfaction. Since the publication of the original EPA guideline in 2015, the field has advanced considerably in its theoretical foundations, evidence base, and institutional support. This updated guideline addresses recent developments and challenges in research, policy, and clinical practice.

**Methods:**

The guideline is based on a systematic meta-review of 15 systematic reviews and evidence-based guidelines on cultural competence training in psychiatric care and mental health. The evidence was synthesized to identify key concepts, educational components, implementation strategies, and current debates.

**Results:**

The review comprises four sections. First, it examines core elements of cultural competence training, emphasizing cultural humility, structural competence, and perspectives from the social and cultural sciences. Second, it provides practical recommendations for curriculum design, including the potential role of digital learning tools. Third, it addresses critiques of cultural competence, particularly concerns regarding essentializing and potentially racist interpretations. Finally, it explores strategies for integrating cultural competence training into existing psychiatric services, highlighting both individual and organizational factors that support implementation.

**Conclusion:**

Cultural competence benefits not only migrant and ethnically diverse populations but all patients and healthcare professionals, including members of majority cultures. Successful implementation requires cultural sensitivity, critical self-reflection, and sustained commitment at individual, organizational, and system levels to achieve meaningful and lasting improvements in psychiatric care.

## Introduction: cultural competences as a critical field

Proven to improve quality of care, effectiveness, and patient satisfaction, cultural competence trainings are becoming more commonplace in psychiatric practice in Europe and worldwide. Cultural competence can be defined as the ability to recognise and sensitively address diverse cultural factors in the therapeutic interaction between the therapist and the patient, seeing both in a dynamic web of interlinking cultural values, beliefs, practices, and privileges. Cultural competence training in psychiatry and mental health care can be characterised as a meta-theory or process of continuous learning and reflection, in which health providers are becoming increasingly aware of both their own and their patients’ often fluid culture(s) at stake during health service provision. Trainings usually aim to develop skills, knowledge, and attitudes to effectively address diverse cultural and linguistic needs of international or diverse patient groups, including but not limited to migrants [1, 2]. As such, cultural competence trainings are widely regarded as “good clinical practice with the goal that all patients, especially those from minority groups, feel acknowledged and supported” ([2], p. 431). For a detailed definition and additional references on the literature of cultural competences pre-2015, we refer the reader to the previously published guideline.

In 2015, an EPA guidance document on cultural competence training [2] already identified key themes and recommendations for cultural competence trainings in Europe. Since its original publication date, the last ten years have not only seen a steep increase in the number of studies on cultural competences in psychiatry as indicated by the numbers of systematic reviews on this topic [3–8] but also see sustained interest and ambition in education and training to respond to the cultural diversity of patients; relevant conceptualisations of frameworks include “cultural sensitivity” [9], “cultural responsiveness” [10] or, more recently, “cultural humility” [11]. Cultural competences continue to present themselves as a relevant and conceptually mature tool for training and practice. The last decade, however, has also brought about a number of demographic, geopolitical, social, and institutional shifts that colour the social, political, and institutional landscape in which cultural competence trainings are embedded. In this light, cultural competence frameworks have also been subject to critical feedback both from within and outside the psychiatry community, highlighting that cultural competence trainings might in fact be *achieving the opposite* of their intention: simplifying, essentialising, or stereotyping culture to such an extent that it might be considered examples of institutional racism [12–14].

To sustain the relevance of cultural competence training, in this updated and newly written EPA guidance, we actively engage with this criticism and concern, framing cultural competence as a cornerstone not only towards good, ethical, or patient-centred clinical practice but also towards mental health equity [4].

## Embedding cultural competences in a changing landscape

### Global perspectives on migration and mental health

In recent years, clinical practitioners have observed an increase in international patients, some of whom are forcibly displaced, undocumented, refugees, or asylum seekers, with complex health needs and impeded access to health services [15, 16]. Immigration remains a central determinant for mental health that requires a multi-dimensional, dynamic approach to understanding the patient “in relation to the environmental, historical, sociopolitical, economical, and other structural contexts that contribute to the experiences” ([17], p. 93). It is well known that factors such as poverty, persecution, or violence may play a role in migration, with other factors like war [17] and climate change receiving more attention recently [18]. In the literature, trauma as an integral part of migration trajectories of complex socio-political events [19] has been linked to culturally competent care (see section Cultural competence as a structural matter of concern).

With a general shift towards the Right in terms of European asylum and health politics, severe cuts or altogether withdrawal of time-sensitive government support for refugees’ mental health care continues to threaten adequate care delivery for groups with traumatic migration trajectories. This means that in places like Germany, migrants’ mental health needs continue to be unmet [20] while cases of forcibly displaced people with complex trauma needs are only likely to go up [21]. Considering demographic changes, migration must also include the lived realities of second or third generation migrants and other long-settled minority groups, some of which may not self-identify as migrants but are nevertheless racialised. In the long run, ignoring migration as a determinant for mental health poses an immense strain on the health and social care system as a whole.

But trends in migration are also interlinked with other social trends that impact mental health outcomes of migrant communities: For instance, rising economic precarity and funding cuts across Europe imply adverse consequences on health, such as a decline in social connectedness, precarious housing and jobs, and a general increase in inequality [22, 23]. Moreover, an increase in politically motivated aggression and violence may be linked to adverse mental health outcomes [24] while an increase in racism poses threats to the mental health of racialised communities [21]. The wars in Ukraine and Israel/Gaza pose complex challenges to both patients and mental health care and practice, with adverse effects likely to span generations [25, 26, 27]. These political developments may themselves be regarded as cultures of radicalisation and fear, that, in turn, may culturally condition cultural competence training. However, some authors remark that political discourses on issues like “hyperdiversity” should not overshadow the broad spectrum of other culturally relevant clinical dilemmas [1, 3].

### The politics of psychiatric care across Europe

Policy makers in various European countries are responding to the change in migration-related health needs in diverse ways: Scandinavian countries, which have, historically speaking, been more culturally homogenous, for instance, have focused on adequate trauma response networks. While over the last few decades in Sweden, an increase in health care training focusing on cultural and social determinants of health can be observed [28]. In Italy, Germany, the Netherlands, Spain, and France, teams have been set up for intercultural competence training. In Germany and the Netherlands, psychiatrists aim to implement culturally sensitive services in public mental health services – noting here, however, a lack of support and funding from governments [29].

In a recent survey amongst psychiatry trainees in Europe, [15] found that only slightly more than one-third of trainees felt confident in working with forcibly displaced persons, such as refugees. The authors link this subjective incompetence to a lack of cultural competence training opportunities across Europe, despite trainees indicating a great motivation and willingness to learn more about culturally sensitive care. For instance, even though culturally sensitive care is universally recommended in the United Kingdom, only 19% of trainees have received appropriate training. Turkey and Ukraine were amongst the countries with the highest rate of received training, with roughly half of trainees having taken part in cultural competence trainings. This study points towards a large gap in the awareness and provision of cultural competence training in the European region and underscores the importance of authoritative and harmonised guidelines for cultural competence training across Europe [6, 30].

But training may not only benefit patient populations: with a 60% rise in migrant health workers between 2010 and 2016, according to WHO data [31], including mental health professionals, cultural competence training may also be relevant as a diversity and inclusion measure on an intra-professional level in a variety of health care institutions, for instance, in the hospital [32].

### Overview

In this meta-review, findings are presented in four parts: (I) This guidance first provides an overview of key elements of cultural competence training, with cultural humility and structural competences [33] as two critical additions to the previous guidance. (II) On the practical side, training tools are reviewed, including a critical engagement with the limits and opportunities of the cultural formulation interview (CFI) and use of interpretation and digital services, and how these may play into curriculum design. (III) On a critical level, the guidance reviews the theoretical engagement of critical competences and stresses intersectionality, racism, and *critical Whiteness* as key theoretical components to productively engage with critical challenges. (IV) On the level of implementation, this guidance embeds previous considerations into an ecological approach, focusing on the importance of collaboration and holistic integration of cultural competences into existing local infrastructures, organisational settings, and models of care. On the basis of this review, a conclusion offers a critical call to action.

For ease of reading and to not get lost within the methodological and analytical detail of this review, the guideline starts off with recommendations.

## Recommendations: cultural competences need to push for nothing less than structural change

As we look into the next ten years, it is clear that there is work to be done to integrate cultural competence into all modalities of training, addressing all professional groups and career levels across all clinical and public health settings relevant to psychiatry. There must be a shift that patients are the only group benefiting from cultural competences, cultural competences are relevant for everyone, including staff members of majority cultures. Patients with “diverse cultures” must extend the notion of “migrant” and consider individuals and communities with a variety of cultural experiences, including those in prisons, military personnel, and people with intellectual disabilities [34].

Given the evidence and conceptual nuance apparent in our review, the following elements need to be added to already existing recommendations:

### For institutions

- **Critical reflection:** Institutions need to critically grapple with and, if needed, transform their own cultural norms, values, and assumptions underpinning psychiatric care structures.
- **Strategic pan-European alliances** are vital in building, sharing, and sustaining training infrastructures for culturally competent psychiatry in all European member states.
- **Monitoring:** Monitoring the number of culturally sensitive care facilities in order to assess demand and develop further appropriate service units. Monitor health outcomes and quality of care on the basis of intersectional markers and avoid oversimplification of complex and diverse patient groups like “migrants.”
- **Mental health equity** needs to be a shared vision and goal across all interest groups.
- **Education:** Establish cultural psychiatry as an integral part of training, starting from undergraduate degrees.

### For management in mental health service delivery

- **Monitor:** Geopolitical events and demographic trends (both nationally and locally) need to be carefully observed and responded to, e.g. translating information into a new language or adapting consultation hours for mothers.
- **Represent and recruit:** Recruiting, upskilling, and promoting an international workforce can help cultural representation and diversity from the provider’s side, but it is crucial to respect health care workers’ rights as well as carefully review ethical standards of recruitment agencies.
- **Leads assigned:** Responsibilities to implement and manage cultural competence trainings need to be clearly established; time as well as financial resources need to be freed up accordingly.
- **Quality assurance**: Review and revision of already established tools for cultural competences, such as the CFI, need to be critically reassessed against a decolonial backdrop.
- **Knowledge transfer:** Evidence on specific cultural competences needs to be transferred between research, practice, care, policy, and teaching in a time-sensitive manner, including professional perspectives from different national contexts.
- **Respond to critical discourse**: Cultural competence trainings need to assess the risk of stigmatisation; cultural humility and structural competence should enhance or accompany the cultural competence curriculum.
- **Innovate for diversity:** Employing peers and co-design, digital technologies, and family-friendly, diversity-oriented working conditions can help innovate and diversify cultures of care.
- **Network:** Find alignment and coordinate between existing services, if needed, across departments, facilities, professions, and between patients, family members, patient groups, civic society organisations, and other institutions – both nationally as well as across Europe.

### For individual service providers

- **Ask to learn:** Clinicians and other staff need to push for their right to be skilled up and regularly supervised on cultural competences.
- **Practice cultural humility:** Individuals need to constantly reflect on their own assumptions and look at patients as experts of their own experience.
- **Step up:** Support peers in learning about cultural competences, consider becoming a trainer (e.g. through a train-the-trainer scheme) and aim for maximal multiplication of learning.
- **Allyship:** Become aware and advocate for patients’ health rights (e.g. right to suitable interpretation), report individual shortcomings and any form of discrimination to the appropriate departments.

## Development of the guidance document

Over the last ten years, the scope and scale of literature studying the design, implementation, quality, or effectiveness of individual cultural competence training in psychiatry have become extensive. Thus, rather than reviewing too large a number of primary studies, recommendations of this guidance document are based on a systematic meta-review, in accordance with other updated EPA guidance documents [35]. A meta-review, or review of reviews, is a systematic overview of meta-analyses, systematic reviews, and evidence-based clinical guidelines, providing both a meta-theoretical overview on the topic of cultural competence in psychiatry, as well as high levels of evidence, presented here in a narrative synthesis.

### Method

The development of this EPA guidance on cultural competence training adhered to the standardised methods defined by the European Guidance Project of the EPA [36] and is based on a systematic literature search performed according to the Preferred Reporting Items for Systematic reviews and Meta-Analyses (PRISMA) guidelines [37].

The literature search was conducted on 16 March 2025 on three electronic databases (MEDLINE and EMBASE [via Ovi]) and PsychInfo) using the following search string for MEDLINE: (Cultural competence [Title] OR cultural competences [Title] OR cultural competency [Title] OR cultural competencies [Title] OR (transcultural [Title] OR trans-cultural) OR (crosscultural [Title] or cross-cultural [Title]), OR (intercultural [Title] OR inter-cultural [Title]) OR cultural [Title]) AND (training [Title] OR education [Title] OR guidelines [Title] OR skills [Title]) AND (psychiatry [Title/Abstract] OR mental health [Title/Abstract]).

The starting date of the systematic search was 2015, the date of the original publication.

Additional manual searches were conducted on PubMed and Google Scholar using slight adaptations of the search string of the original guidance paper. Reference lists of included works were manually inspected, and an expert panel shared other possibly relevant articles with the author team.

### Selection procedure

The following criteria were applied for study selection.

Inclusion

- Systematic and scoping reviews
- Unsystematic, narrative review and conceptual guidance with clear search terms and a low degree of selection bias
- Reviews and conceptual overviews with a central focus on cultural competence in the context of psychiatry or mental health care and training
- Articles published in any European language (if the abstract was available in English)

Exclusion:

- Duplicates, comments, editorials, case reports/ case series, theses, proceedings, letters, short surveys, and notes
- Articles not referring specifically to cultural competences (e.g. just referring to “multiculturalism”)
- Unavailable full text
- Articles that do not meet inclusion criteria (e.g. empirical studies on a single cultural competence training framework)

All documents were independently screened, data extracted by two reviewers, and discrepancies discussed with a third reviewer.

**Figure 1.**
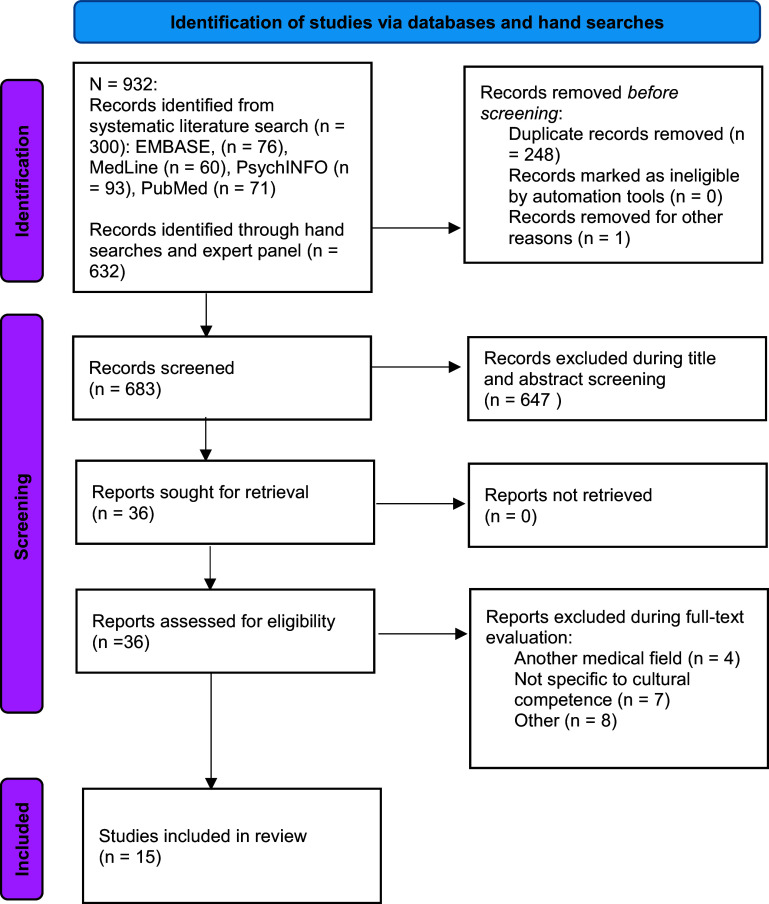
PRISMA flow chart. Figure 1. long description.

The PRISMA flow chart (Figure 1) shows the selection procedure.

### Grading of evidence

Reviews were systematically graded through the SIGN checklist for systematic review and meta-analyses (www.sign.ac.uk/using-our-guidelines/methodology/checklists/) as recommended by the EPA guidance [36] by three reviewers. All disputes were discussed within the author team.

### Included studies

In total, 15 studies were included in the systematic review.

Ten narrative, qualitative, non-systematic, or systematic reviews were included [1, 3–8, 34, 38, 39], each of which included recommendations, tools, or guidelines aimed at practitioners. Of these, one review [39] provides a specifically authoritative review and guidance, as an update of a previous version [40].

Results also include five conceptual articles that took a critical stance towards future directions of cultural competences in psychiatric training [14, 17, 41–43].

To contextualise these results, additional studies, guidelines, and articles are cited in the remainder of this document, most of which are published after 2015. As an example for a relevant exclusion: neither the APA nor the UEMS guidelines on working with diverse and multiple cultures matched our inclusion criteria, as they were not specifically concerned with cultural competences. Relevant elements of these guidelines were still included.

Our review also stands in context with other important reviews that did not meet our inclusion criteria (mainly because they did not consider cultural competences specifically for mental health service provision) but are nevertheless important. These include reviews of more general psychiatric training programmes for ethnic minority groups and migrants [29, 44], a scoping review on cultural competences in refugee service settings [45], reviews of cultural competences in medical education [46, 47], a review of cultural competence training programmes in unspecified health care settings [48] and a review of antiracism training in psychiatric care [49].

### Findings

#### Elements of Cultural Competence Training

Elements of cultural competence trainings vary widely in terms of content and learning aims. In a systematic review of 37 cultural competence trainings for mental health providers, Chu and colleagues [3], for instance, found that 64.9% of all reviewed trainings focus on race/ethnicity, followed by sexual orientation (45.9%) and general multicultural identity (43.2%). Fewer curricula explored other cultural dimensions; religion features in 16.2% of trainings, immigration status in 13.5%, and socioeconomic status in 13.5%. Information on sociocultural background was, however, a key component in 89.2%, followed by identity (78.4%). More critical elements like discrimination and prejudice were featured in slightly more than half of the trainings (54.1%). They also found that cultural attitudes were the most frequently assessed training outcome (89.2%), followed by knowledge (81.1%) and skills (67.6%).

The following components, concepts, and qualities have been identified as relevant in developing cultural competences and are presented here in an updated comprehensive overview:

##### Cognitive cultural competence

Cognitive cultural competence, otherwise known as “knowledge,” involves awareness of the various ways in which culture, immigration status, and processes of racialisation impact psychosocial development, psychopathology, phenomenology of illness, and therapeutic transactions. For instance, it has been shown that the experience of depression varies between cultures and can show itself as feelings of sadness, guilt, shame, or persecution, and that it can be somatised in different ways [50]. Diverse experiences may not only be experienced but also expressed in different ways, for example, through different idioms of distress. Idioms of distress may include narrative, symbolic, and non-verbal ways of expressing ways of referring to a specific kind of psychological experience, like depression. Explanatory models may differ across cultural groups, for instance, in some cultural traditions, psychotic symptoms may be interpreted in relation to spirits or other seemingly supernatural forces [27].

It is not only symptom presentation that varies, but the way that patients can make themselves understood to clinicians also relies on their understanding and experiences of (mental) health terminology and willingness to talk about themselves in a language that can be interpreted as pathologising and stigmatising. A cultural perspective on medical knowledge involves an understanding that psychiatric conditions are sociocultural constructs as much as they are medical conditions. Diagnosing personality disorders in different cultures, for instance, requires an awareness of acculturative tensions and clashes which, depending on other personality traits, can affect different types of behaviours, which may seem unusual or excessive but are not necessarily indicative of psychopathology [8, 51]. The ICD-11 diagnostic requirements for personality disorders authoritatively underline these studies by stressing the requirement for “knowledge of normative personality function for the sociocultural context, variations in cultural concepts of the self, and evidence for consistent traits and behaviours across time and multiple social contexts” [52]. The ICD-11 gives an example that children in more collectivist cultures might be perceived to be dependent in their attachment styles in comparison to more individualistic cultures, whose cultural inclination towards self-involvement may, in turn, be read as narcissistic by collectivist cultures.

##### Cultural attitude

Cultural attitudes are unquestioned assumptions and beliefs that colour practitioners’ knowledge, views, and behaviour. Intercultural work requires psychiatrists to challenge their own perceptions of “reality,” explore their own cultural identity, conventional wisdom, prejudices, and biases, reflected in the way they look, their religion, life habits, title, or possible sociocultural message linked to their name. Cultural attitude can also be seen as a moral, social, and local reflection of one’s position and the ability not to assume that one’s position is “normal” [48]. One important aspect is that of cultural counterference, which can be described as the clinician’s thoughts, feelings, and attitudes that come up in relation to a patient’s perceived cultural “otherness.” Intercultural skills in this context can be understood as building a therapeutic relationship with a “culturally different” patient, and the ability to adapt diagnosis and treatment in response to cultural differences between the psychiatrist and the patient [53, 54]. (Trans-)cultural competencies are important to ensure the transferability of psychiatric expertise between different cultures and national standards.

##### Cultural sensitivity

Cultural sensitivity implores clinicians to create an open and safe environment in which patients feel sufficiently comfortable to explore difficult and painful ideas and emotions [55]. Cultural sensitivity can go hand in hand with cultural empathy and insight, the ability to actively listen to a patient’s experiences of illness within the context of their culture and acculturation processes. As an example, cultural sensitivity might pay attention to how patients’ transnational and multi-generational migration experiences can lead to “cultural bereavement” [56] – a perceived loss of the familiar language (especially colloquial and dialect), attitudes, values, social structures, and support networks that occurs when moving from one cultural and social setting to another. Cultural sensitivity might then require an open narrative inquiry into roles of religious or natural symbols and images, functions of elders, and cultural rites and rituals [50]. In consequence, on the patients’ side, cultural sensitivity can create cultural safety, the feeling that one’s culture is the experience of feeling that your cultural identity is recognised, respected, and nurtured.

##### Cultural humility

Another concept that has grown in prominence over the last ten years is that of cultural humility, which can be understood as the de-centring of the health care professional as the only or “real” expert in the room. As such, it is an integral part of patient-centred cultural competence training [29, 39]. Various aspects are involved in showing cultural humility: (i) a continuous commitment to self-reflection and self-critique, (ii) a conscious addressing of power imbalances between patient and clinicians as well as different occupational groups within the wider care sector, (iii) the effort to meaningfully engage in non-paternalistic partnerships [17]. On the side of the supervisor, this might require deferring to a trainee’s specific skills, knowledge, and immigration experiences as long as that is not done in a tokenistic way [39].

Asking questions with a curious mind, seeking to engage with doubt and uncertainty, and not settling for simple explanations are other hallmarks of cultural humility. This decentring must go hand in hand with a critical reflection of “Western” medicine and an openness to value the wisdom presented in communities and ancient traditions, including religion and spirituality [57, 58].

##### Structural competence

Another addition to the understanding of cultural competences is that of structural competences. Some commentators go as far as suggesting a complete shift from culture to structure in competence frameworks [41, 59, 60]. Structural competences require a political commitment to working towards human rights, social justice, and health equity by tackling the root causes of poor mental health and wellbeing. Most importantly, they require clinicians to develop an understanding of the social determinants of health, for instance, immigration status and housing.

##### Theoretical understanding

A final element of cultural competence training is grappling with theories and concepts that have largely evolved in the Social Sciences and Humanities but also in health-related disciplines such as Medical Anthropology, Public Health, or Social Epidemiology [61]. These include theoretical understandings of racism and intersectionality, both laid out in section Critical engagement: cultural competencies in dialogue of this guidance.

#### Curriculum design and training tools for cultural competences

Organisations like the World Psychiatric Association (WPA) stress that incorporating cultural competency in education is a *must* for trainee medical experts in psychiatry [62], and a non-negotiable for high-quality training. A number of recent reviews show, however, that the nature of these trainings is heterogeneous in terms of the variety of provider groups, patient groups, outcomes, resources, scope, evaluation, and integration into different clinical aspects [6, 7, 29, 46, 63]. To better understand how to best integrate cultural competences within institutional settings, first, this section lays out the importance of collaboration and then introduces four tools integral to cultural competence training: CFI, interpreters, cultural brokers, digital tools, and other innovation tools.

##### Cultural competences in the curriculum: a collaborative process

In a scoping review on the topic of cultural competences in medical education, Gruner, Feinberg [64] identified that the most common learning objectives included access to care barriers, social determinants of health for refugees, cross-cultural communication skills, global health epidemiology, challenges and pitfalls of providing care, and mental health; each of which requires a systems approach to teaching and thinking.

In another systematic review of cultural competency training for psychiatry residents and mental health professionals [7], the authors draw out, compare, and analyse various training models in cultural competency training across their 14 included studies, grouping them as: active/passive/mixed; group training/individual training. They find that in general active methods of training, including for instance documentary film, video demonstrations, case vignette discussions, and various case and group formats, as well as mixed training, showed better outcomes than more passive learning methods, such as reading or lectures.

Chu and colleagues [3] found in their systematic review that the most common types of instructional categories in training are lectures (89.2%), discussions (86.5%), or assignments and exercises (e.g. presentations or privilege walk, 59.5%). More hands-on, practical instructional tools were much less common, though (e.g. clinical experience: 16.2%; modelling: 13.5%).

In terms of designing the cultural competence curricula, Kirmayer et al. [39] stress that cultural competence training needs to be ongoing and recommend a combination of theory lectures and rotation (of 3–12 months) in three major domains: direct patient contact, consultation with referring clinicians, and outreach to community referral sources, including a mix of cultural consultation, community consultation, short-term treatment of refugees and survivors of torture, child and family assessment and interventions, as well as cultural psychiatric research.

The Canadian Psychiatric Association shows useful core competencies and didactic principles in their updated guideline for cultural psychiatry. In comparison to an older version, where Kirmayer, Fung [40] identified the following as core themes in a cultural psychiatry curriculum: culture and health, culture, illness and psychopathology, culture in clinical practice, and culture and health care policy, services and systems; now it is stressed that cultural competences cannot be readily taught in a classroom but are rather experiential learning and a process of reflection, often in a group setting [39] and for all career stages, not only residents [34].

Teaching competence among educational leaders and peers is a key prerequisite for the effective delivery of cultural competence training. For instance, culturally competent didactics should include elements of peer learning and take into account different learning styles, some of which may be due to different cultures of learning and education, as well as approaches in different disciplines [65]. One such training modality may be the interdisciplinary case discussions as outlined by Rousseau, Johnson-Lafleur [66], here the authors explain how they critically implemented the CFI into their everyday practice. Culturally competent clinical supervision needs to integrate and continuously reflect upon learnings in everyday clinical practice on a regular and routine basis [17, 38]. Training in cultural competences must be seen as a continuous and never-ending process.

Recent publications have underlined the importance of pairing training in cultural competences with awareness and anti-racism training built into an ethics of accountability and continuous reflection on power differences between different social and cultural groups [14]. Cultural competences may also be implemented in a larger advocacy framework, whereby “doctors can and must speak on behalf of all those who may not be able to speak for themselves” [67], again highlighting the ethical importance of using one’s privilege for good.

##### Cultural formulation interview

The CFI of the DSM-5, originally developed in 2013 by the American Psychiatric Association (APA), offers a basic approach of understanding the cultural context of a patient’s experience of illness, this being essential for effective diagnostic assessment and clinical management, and has been reviewed and tested widely [68]. Since then, it has been translated and implemented in a variety of cultural settings [68, 69]. Research indicates that the CFI’s open-ended questions can establish a patient-centred approach, centring patients’ opinions, perceptions, and experience, thereby allowing them to better build their own models of illness [70]. It is a vital element to decrease or avoid mistrust and build a strong therapeutic alliance [70]

The CFI consists of an interview protocol with 16 semi-structured questions, taking around 20 minutes, with a training time of around one hour [69]; the questions refer to four domains of assessment: Cultural Definition of the Problem, Perceptions of Cause, Context and Support, Cultural Factors Affecting Self-Coping and Past Help Seeking, and Cultural Factors Affecting Current Help Seeking [71].

Since the publication of the original guidance document in 2015, the CFI has been implemented and tested globally [70] and has proven itself as a useful framework for culturally sensitive psychiatric care in various settings [69, 70, 72]. Using the CFI, psychiatrists may obtain information during the mental health assessment about the impact of culture on key aspects of the patient’s clinical presentation and care, making it a critical tool to master the cultural formulation of clinical cases [34]. The CFI has also been found to facilitate the identification of depression diagnoses amongst migrants [73].

In contrast to previous versions, the CFI in the DSM-5 includes more sociologically minded and structurally thinking question prompts like “Has anything prevented you from getting the help you need – for example, cost or lack of insurance coverage, getting time off work or family responsibilities, concern about stigma or discrimination, or lack of services that understand your language or culture? What got in the way?” In successful implementations, specifically trained staff, like social workers, assess these more structural stressors and, if desired, refer patients to transportation vouchers, housing, food stamps, or vocational training [74]. Assessment by parties other than the clinician can be a useful way to make the implementation and training of the CFI feasible.

However, the CFI has not gone uncriticised: a qualitative study in Denmark [75], for instance, found that mental health professionals experienced distance, “othering,” and discomfort in the encounter with patients, critically highlighting a potential risk of stereotyping. Other critical reviews [69, 76] suggest that the CFI may be more difficult to conduct with patients who have severe symptoms and that, worse, it may also lead to racist stereotyping [12]. Understanding psychopathology and formulating psychiatric diagnosis as part of the CFI could be facilitated by a multi-dimensional approach, more than by a categorical approach [27, 77, 78].

Practices and tools for cultural competences, such as the DSM-5 CFI or case-based learning, have been deemed problematic in that they may lead to stereotyping, meaning that if a specific experience was found to be true for one patient of a specific culture, that truth is then applied to other patients believed to belong to the same culture [12, 29, 50, 69]. In their study on implementing the CFI in Canada, Rousseau and colleagues [66] posit that interdisciplinary case discussion seminars are a useful technique to avoid stereotyping and sensitise CFI-interviewers towards in-group differences and multiple levels of diversity (individual, disciplinary, and interinstitutional), underlining that cultural formulation is always relational and possibly just a snapshot taken in a moment in time.

##### Use of interpreters and cultural brokers

Collaboration with trained interpreters, cultural brokers, or transcultural mediators [79] is an integral part of cultural competences. Clinicians should be trained in cross-language interviews and when interacting with the interpreters and be aware that there is no guarantee for complete understanding. Patients need to be empowered when they do not have proficiency in the languages spoken by the clinicians [80]; doctors need to act as advocates helping patients enforce their legal rights to interpretation services.

Linguistic abilities are a massive barrier to accessing care; for instance, the communication of distress in the face of language barriers can be a significant reason for non-engagement, increased levels of dissatisfaction, and drop-out. The idiom of distress in which patients communicate with psychiatrists can vary considerably across cultures and languages. We know that many languages do not have equivalent words to describe various mental disorders. For example, the word and notion of “depression” do not exist in all cultures, even though sadness, unhappiness, and other symptoms can be described and verified. Presenting with somatic symptoms, like sleep disturbances – a common symptom for depression in the Black community [81] – may delay diagnosis and treatment and can carry with it the risk of unnecessary clinical investigations.

Another challenge in successfully working with interpreters is the lack of common criteria and standards across and within Europe, and the fact that hiring agencies have no incentives to enforce those requirements that exist [15]. And although service providers are required by law to provide sufficient interpretation services, interpreters of more unique languages or dialects are often hard to get by and require longer waiting periods. Too often, their cost is not reimbursed, even though they are very cost-effective in the long run [82]. Moreover, in out-patient care, the extra time required for interactions with qualified interpreters is not compensated, and interpreters, especially those for less common languages, are often booked out, requiring long waiting.

Although ad hoc solutions like non-professional translators (such as family members, hospital staff members) or the use of open access translation tools, like Google Translate, can be very helpful in urgent situations, relying on such informal sources can have a negative impact on medical treatment due to erroneous translation in the form of omissions, additions, or indeed changes to the initial message, not to speak of the emotional strain on those involved. Working with language interpreters and cultural brokers, if available, seems to come with clear advantages and improved treatment outcome in terms of quality and patients’ satisfaction [83].

In practice, however, Frankova and colleagues [15] challenge that working with interpreters is universally good. In their survey of clinicians working with forcibly displaced patients (FDPs), they found that although half of the respondents felt confident working with an interpreter, paradoxically, those who felt most confident working with FDPs felt less confident working with interpreters, although reasons for this and its effects on quality of care remain unclear. Another notion is added by Kirmayer et al. [39] who argue in their guidelines on cultural competences, working with professional interpreters and cultural brokers may also require tolerance for ambiguity and uncertainty, where professionals have to *un*learn a vocabulary of mastery, precision, and efficiency in favour of listening [38].

##### The role of (digital) simulations, technologies, and artificial intelligence

In psychiatric training, simulations with actors in real-life or virtual classroom settings may play an important role in becoming culturally competent with patients from diverse cultures. In a review about simulation training in psychiatry, Piot et al. [84] highlight that simulation may help trainees to grapple with the cultural-historical meaning-making processes in how mental disorder is displayed in various person-centred care settings. Virtual patients and other interactive computer simulations may also offer innovative training opportunities in significantly improving confidence in cultural competencies [85, 86]. Telehealth consultations may also help to improve cultural safety in that they facilitate access to skilled interpreters and culture brokers; in the context of Indigenous care, though, Terrill and colleagues [87] argue in a scoping review that face-to-face communication remains irreplaceable.

##### Other innovative tools

More innovative models could include cultural exposure through resident and faculty international medical graduates, rotations across different European and non-European countries, and cultural exchange of professional staff with other international medical schools [51]. Although it might appear common sense to foster a culture of innovation in cultural competence training, the literature currently lacks a more thorough engagement in pedagogies and creative approaches to innovation in cultural competence training and development.

#### Critical engagement: cultural competencies in dialogue

Notwithstanding the richness in evidence and intellectual engagement, criticism towards the appropriateness of cultural competence trainings remains vocal. This section addresses this criticism more specifically and offers ways forward.

All included studies in this review were in some form or another critical of the word “cultural” and assumptions carried in the understanding of “culture” [1, 41]. The way that culture is sometimes talked about in the literature on cultural competences might suggest that culture is something uniform that a person, often a migrant simply “has” or “is.” This is problematic on a number of levels:

##### Health as culture

Importantly, all reviews underline that culture should not be read synonymously with “ethnically different”; in fact, everybody has a culture, and developing an awareness of one’s own beliefs, values, assumptions, and taken-for-granted practices and rituals is core in practicing cultural humility. But it is not only individuals who have different cultures; the institutions in which they work are also deeply cultural.

Culture needs to be understood as a dynamic, relational, fluid, and constantly contested process, where one’s culture is dependent on context, time, and place [41]. Acculturation processes have been identified as one way of addressing the complex interplay between different cultural dimensions and processes of adapting others’ cultural norms, values, and practices over time. A criticism of acculturation theory, however, is that it does not factor in the dimension of power.

The UEMS’ understanding of culture, for instance, also emphasises the difference between different therapeutic cultures and their understanding of health, illness, and recovery [88]. Different health beliefs are tied to different values, traditions, and also reflect in the ways that patients understand and communicate their symptoms, and psychiatric services are shaped and delivered in various European countries [89]. It is therefore important to consider cultural practices within dominant groups and consider health professions within psychiatry as their own unique culture, built on a set of assumptions, values, and beliefs about who can be deemed “healthy,” “ill” and in need of help. Following the recommendation by the UEMS, a way forward for psychiatric care in Europe must be “recovery-focused and trauma-informed with a culture of respect for human rights” [89] (Box 1).

Box 1. Health as a culture

Health as a culture also implies a critical reckoning with the fact that psychiatry as a discipline has been institutionalised as a White and Eurocentric practice closely entangled with colonial practices and incarceration systems, with the goal of keeping the “other” or the “insane” away from “us”; necessitating a careful consideration of the history and negative image of psychiatric practice [32, 44, 59, 90, 91], as also outlined by previous EPA guidance [92]. In the context of cultural competences, a more radical approach calls for decolonising health institutions by learning about and integrating alternative or Indigenous healing practices (as long as they are safe); here the aim is to recognise and repair harm historically done to non-White communities. In a European context, this might also involve reckoning with the lasting cultural trauma and institutional remains of genocidal psychiatric violence, for instance, in the shape of scientific experiments conducted during National Socialism. Giving voice to survivors and witnesses, critical work in medical archives, and an active memorial culture in health institutions are steps towards taking responsibility and decolonising psychiatric care (see, for instance, [39, 91]).

Other examples include recent anti-fascist statements from the APA and the Royal College of Psychiatrists [93] that offer apologies to communities that were historically stigmatised and abused in the mental health system.

##### Intersectionality: Culture as Identity

Cultural competence is often cited in the context of providing mental health care for migrants. In this context, “culture” is sometimes used synonymously with “race,” ethnicity, nationality, or language, often different from that of the majority culture [59, 90, 94]. Not only is this problematic, but it also essentialises and therefore stigmatises a highly diverse set of patients as “migrants” [12]. It is also problematic in that it neglects other dimensions of culture, such as age, gender, family values, regionality, and religion – all of which are an integral part of understanding culture as dynamic [95].

The Accreditation Council for Graduate Medical Education (ACGME) (see table 1 in [96]), for instance, moves away from seeing migrants as the only relevant patient group with a “culture” or as beneficiaries of cultural competence training. Instead, their framework differentiates between different age groups as well as other identity markers such as socioeconomic status, gender, LGBT issues, religion, and disability. To understand how culture is political, reviews have highlighted the importance of the concept of “intersectionality,” specifically given that current training tools in cultural competences are largely void of these intersectional dimensions [34].

An intersectional approach to cultural competence training would, for instance, take into account how migration status or processes of racialisation connect to gender, sexual orientation, income, education level, or age [97, 98].

##### Racism: cultural competences cannot be apolitical

A key criticism towards cultural competences has been that an over-emphasis on “cultural differences” in approaching patients with complex health needs possibly over-simplifies or neglects other complex bio-social, political, economic, and environmental factors to good mental health [59]. Reducing these holistic factors to cultural affiliation alone might mean missing other important areas of intervention and might leave deeper-seated power imbalances or inequalities unaddressed [94].

A central power dimension often met with defence is racism. As Jarvis et al. [12] argue, many psychiatrists have not been trained to see *structural violence* as part of their clinical landscape, and thus fail to recognise racism as a force that shapes diagnostic outcomes, access to care, and therapeutic relationships. When racism is framed as systemic rather than interpersonal, it demands not just awareness but accountability and change [12, 34, 91].

Racism has received increasing attention by the psychiatric community, stressing here that it is no longer enough to not be racist, psychiatrists must be actively anti-racist. This may be achieved by considering processes of identity formation, including Othering, more research on anti-Blackness, as well as critically interrogating Whiteness within mental health care. In general, recent publications have highlighted that theorisation and conceptualisation of racism in Europe are still in its infancy [99, 100] (Box 2).

Box 2. Forms of racism in the context of care

Interpersonal racism: includes both direct verbal harm, as well as non-verbal harm, such as eye-rolling, (micro-)aggression (like unwanted hair touching), (micro-)invalidation (like saying: “it is not a big deal”), or repeated inconsiderateness (e.g. misspelling or mispronouncing a foreign name or asking “where they are really from”).

Systemic racism: states that entire systems, such as the medical education, are entrenched with social orders and norms that can be traced back to a false assumption that White people are superior, should generally be shielded from harm or distress, or deserve more or better psychiatric care.

Structural racism: can be understood as the scaffolding of these systems, it includes policies, laws, care infrastructures, or knowledge production processes. Structural racism also shows itself in how fast and easy it is for someone to access appropriate care; racialised patient groups often have to wait longer for an appointment or jump through extra hoops before appropriate treatment can begin.

In the context of central Europe, anti-racism in psychiatric care might include not only a deep engagement with non-White communities, such as the African Diaspora, but also with the mental health experiences of racialised communities that may, judging from their complexion or skin tone, for instance, be present as White. Examples include Roma and Sinti communities, Jewish communities, or Eastern Europeans (or “Slavic” communities).

#### Cultural competence as a structural matter of concern

To work towards intersectional, anti-racist psychiatric practice requires not only a cultural shift but also a structural shift. We therefore advocate for a holistic integration of cultural competence in transformative change through advocacy [67] and solidarity [101].

##### Integration into structural competences and structural change

In the UEMS’ European framework for competencies in psychiatry, the authors underline the importance of “respecting all other barriers, such as the cultural differences between the countries with regard to how mental health care is considered and funded, neglected role of psychotherapy, culture and family context” [102]; here the psychiatrist can take the role of a collaborator with other social and cultural networks (p.356). The adjoining document “Training requirements for the specialty of psychiatry” by the UEMS specifies this advice with reference to knowledge tests, clinical examinations, and in-training assessment. Importantly, cultural differences are not only to be found between different countries but also, of course, within individual countries, (federal) states, or regions.

On the other hand, institutional cultural competence requires not only the recognition of the barriers that exist to quality care at a systemic, organisational, and institutional level but also the elimination of these [103–105]. The demand to remove barriers to access have more recently been linked to discourses on health equity in psychiatric care [14, 106]. Some of these barriers are relatively straightforward, such as having insufficient professionals who speak the same language as the patient, a lack of access to services via public transportation, restricted opening hours of a centre, and so forth.

##### Integration into trauma-sensitive care

There seems to be a strong case for the usefulness of cultural competences when working with care of FDPs, that is, people who have fled war or torture and are seeking refuge in one of the European countries [15, 107]. This population often presents with complex migration histories alongside psychological trauma, which is itself deeply cultural and sometimes trans-generational; this trauma may be pre- or post-migration [47].

The role of culture in the experience and reporting of trauma needs to be anchored in cultural competence curricula [108] and trauma care alongside a profound socio-political understanding of war and immigration as a social determinant for mental health [17, 107, 109]. For instance, a study for culturally sensitive perinatal psychiatric care in a hospital in France [50] highlight that culturally sensitive clinicians might consider traumatic dimensions in countertransference in order to gauge the impact of war and terror on individual patients. Working with this group of traumatised patients, psychiatrists need to take into account the risk of vicarious trauma, such as a “blank mind” or seemingly false representation of facts, places, and times. Cultural competences then need to be embedded into an agenda of global public mental health and a general understanding of historical and geopolitical processes that inform how trauma may present itself within specific migration trajectories and cultural settings [109].

##### Integration into community-centred care and family structures

Key publications recommend seeing the patient within the context of their communities and family, and considering the patient’s family’s history of (ongoing) trauma [30]. These developments highlight the link between cultural competence training and training in (complex) trauma and community care [109, 110]. Actively involving family members in psychiatric care can be helpful to understand the acculturation process and to interpret subtle cultural differences within groups. Incorporating community elders can also help establish contacts with community associations, the voluntary sector and other actors relevant to delivering psycho-social support to at-risk groups [111, 112].

##### Integration into advocacy and political action

Cultural competence is just one type of competence that is required, next to a host of others cited in the literature on refugee and asylum seekers’ mental health care; for instance, in a scoping review focusing on displaced children and young people’s health needs [6], the authors identify five other key competencies next to cultural competence: (1) knowledge of the complexity of the needs of refugees; (2) use of holistic approaches; (3) ability to work in coordination with others in a child’s network; (4) ability to build therapeutic relationships; (5) seeking feedback. Their findings support other guidelines and statements, such as those of the UEMS [89] and Kirmayer et al. [65] that advocate for a networking perspective and collaboration with civic society organisations, such as housing and employment agencies. Advocacy also entails an active feedback culture that meaningfully involves communities in shared decision making, seeing patients as the actual experts [112]. Involving beneficiaries, such as forcibly displaced people, in co-creation and other patient-centred initiatives is identified as a key pillar for equitable service delivery [47, 108, 110].

##### Integration into organisational settings

But cultural competences must not only happen on the level of the individual but also on the level of the organisation, especially on the management level, who need to take an active stance against discrimination [32]. Anti-racism and anti-discrimination policies and infrastructures must be installed and integrated into cultural competence training. For instance, trainees must be aware of where and how to report suspected instances of discrimination in their specific clinic, organisation, or association. Additional support must be made available for those affected by or witnessing discrimination, all while making their safety and (if desired) anonymity the highest priority.

Awareness should also be central in the way that service facilities and service delivery are designed. The culturally sensitive hospital for instance is dedicated to ensuring culturally sensitive food options (e.g. marking halal meat options), culturally sensitive reading materials in waiting rooms (e.g. avoiding stereotyping children’s books or offering magazines in different languages), access to safe sanitary facilities (e.g. indicating if male cleaning staff is likely to be present in female bathrooms), and a warm and inviting atmosphere (e.g. warm lighting, comfortable seating and culturally sensitive wall art, like abstract paintings rather than people and places). For facilities in countries where English is not the first language, it may be helpful to install bilingual signage and way-markers, all while carefully observing the language barriers and language needs of international as well as domestic patients, some of whose second language may be French. Signage or translations based on images, icons, and simple language may also be helpful. Initiatives like the culturally sensitive hospital [113] are good practice examples for linking cultural competences with diversity and integration officers in the hospital and other (public) health settings [114].

##### Integration into personnel measures

Hiring processes, faculty development, and human resource departments also play a key role in the culturally sensitive clinic. A more diverse staff can aid innovation and is vital for practices of continuous peer learning [39]. Training the trainer initiatives (a core element in structural competence training, for instance) and further learning and teaching opportunities need to be actively supported from a management level. Involving “experts by experience” in community-focussed psychiatric care and linking with voluntary sector organisations are ways to improve the “image of psychiatry” [92], both as a service provider and a possible employer. Building peer-led infrastructures where people usually labelled “patients” can become “providers” (in a wide or narrow sense) can be a crucial step towards inclusion and justice in the clinic. One best practice is the Weddinger Modell, a participatory model originally developed in Berlin, Germany, that aims to reduce coercion and improve safety for patients and staff on the ward by, for instance, employing former patients as peers [115].

##### Evaluation, monitoring, and incentive structures

It is also necessary to build local infrastructure to implement cultural competency training. Organisational change might be achieved through more comprehensive monitoring and evaluation of diversity-related health outcomes [61, 116]. Evaluations are an important component in assuring accountability on an organisational level.

The word “competence” in cultural competence may suggest that deep-seated inequalities can be bridged through simply learning a new skill, implying here that once enough knowledge and tools are acquired, the clinician becomes an “expert” in understanding the complex experiences of others [17]. In clinical studies, competence is often measured in terms of confidence or comfort, which may be unrelated to how respectful, collaborative, and effective psychiatric care across differences actually is. Measuring cultural competence might need to take into account a clinician’s in-session behaviour [90], their self-reflective practices, for instance, during supervision, and their diversity-oriented commitment in social and community life outside the clinic. Recent literature focuses more on the importance of grappling with *in*-competence, in the sense that tolerance of ambiguity, the willingness to listen, and adopting a learner’s mindset, which need to be at the heart of interacting with groups that we perceive to be different from ourselves [39].

Therefore, performance indicators need to be carefully reviewed and checked against established frameworks, such as diversity, equity, inclusion, and belonging frameworks and initiatives [114]. It may be helpful to include more qualitative evaluation measures, such as multi-stakeholder focus groups [47, 108]. Accreditations and other methods of local monitoring can support and enhance the status of the ongoing process of the training [40]. Evaluation practices can be combined with economic and other incentives, although critical concern around tokenism needs to be taken into account. Community-focused and equity-driven incentive structures might include fundraising and donations to local charity organisations.

## Conclusion

We are re-stating that cultural competency is *not* about learning a specific set of skills or language, it is *not* appropriating the cultural practices or adopting the cultural values of a patient, and it is also *not* about the patient integrating into the culture of their host country (if there ever was such a thing as one homogenous culture to begin with). Rather, as previously argued, cultural competences can be understood as a meta-theory and process of continuous diversity-oriented reflection and learning with the aim of improving the quality of diagnostic and therapeutic care. A culturally competent approach of a mental health professional is inherently curious about their own and their patient’s culture(s), aware of the power mechanisms behind these differences, and equipped to reduce different barriers to access for culturally diverse patients.

Cultural competence in mental health care has proven itself to be an important tool in ensuring equitable care for diverse patient groups. This guidance underlined the importance of seeing cultural competences as a continuous process of reflection and learning. This includes facing up to the discomfort of being part of a racist structure within psychiatry and the discomfort of not always being able to know everything about another person’s background. Cultural competence as a process is also political; it asks for a political stance against discrimination, against racism, and for mental health equity for all patients. Responses from European countries rightly differ, but we believe that the core principles as outlined here must be agreed to and employed in further service development.

Looking back at how in only a decade the field of cultural competence scholarship has significantly developed in terms of theoretical rigour, evidence base, and institutional support, we remain hopeful that the future of cultural competence training and scholarship continues to leverage its privilege and influence, embarking on forging new psychiatric cultures of care, curiosity, and, most of all, courage.

## Data Availability

No publicly available data have been used to support the present paper.

## Long descriptions

### Figure 1 Long description

At the top, a blue box states identification of studies via databases and hand searches. The first box below lists N equals 932 records identified, with breakdowns by database and hand searches. An arrow points right to a box detailing records removed before screening, including 248 duplicates, zero by automation, and one for other reasons. Downward, 683 records are screened. A rightward arrow shows 647 records excluded during title and abstract screening. Downward, 36 reports are sought for retrieval, with a rightward arrow indicating zero reports not retrieved. Downward, 36 reports are assessed for eligibility, with a rightward arrow showing 19 reports excluded during full-text evaluation, subdivided as 4 in another medical field, 7 not specific to cultural competence, and 8 other. The final downward box shows 15 studies included in review. The left margin labels the vertical sections as Identification, Screening, and Included.

Navigate back to Figure 1.
